# Bupropion-Associated Delayed Onset Urticaria

**DOI:** 10.7759/cureus.18297

**Published:** 2021-09-26

**Authors:** Nikolas Gutierrez, Sara Malik, Philip R Cohen, Aubrey E Winn

**Affiliations:** 1 General Practice, 1st Marine Division, 1st Combat Engineer Battalion, Camp Pendleton, USA; 2 Feinberg School of Medicine, Northwestern University, Chicago, USA; 3 Dermatology, University of California, Davis Medical Center, Sacramento, USA; 4 Dermatology, Naval Hospital Camp Pendleton, Camp Pendleton, USA

**Keywords:** urticaria, skin, hypersensitivity pruritis, drug eruption, delayed, bupropion, antidepressant, adverse

## Abstract

Bupropion is an oral antidepressant that is commonly used to treat various mood disorders. Its therapeutic mechanism of action results from inhibiting the reuptake of norepinephrine and dopamine. Bupropion is generally well-tolerated and patients usually experience no side effects or mild adverse events. Rarely, hypersensitivity reactions to bupropion, such as urticaria, have been observed. However, the prevalence of delayed onset bupropion-associated urticaria may not be properly reflected in the literature due to misdiagnosis of the condition caused by its atypical presentation. We report a 20-year-old man with delayed onset bupropion-associated urticaria; he had been on bupropion for three weeks and subsequently developed new severely pruritic, erythematous wheals on his abdomen, bilateral flanks, and upper extremities. The clinical features of this rare adverse reaction to bupropion are also summarized.

## Introduction

The novel coronavirus disease 2019 (COVID-19) pandemic has required unprecedented lifestyle alterations and physical isolation that has negatively impacted the mental health and well-being of individuals around the world. Additionally, population-based studies in various countries have demonstrated a significant increase in the incidence of mood disorders, prescriptions for antidepressants, and suicide ideation and attempts since the initiation of lockdowns and restrictions. With the surging rate of antidepressant prescriptions, it can be anticipated that there will also be an increase in observed common and uncommon adverse reactions associated with these medications, highlighting the need for continued reporting of rare adverse reactions [1,2].

Bupropion is a selective dopamine and norepinephrine reuptake inhibitor used to treat various mood disorders. This medication is generally tolerated well with common adverse reactions to include agitation, dry mouth, headache, insomnia, and nausea. Uncommonly, bupropion has been associated with dose-related seizures and increased suicidality; rarely, hypersensitivity reactions have also been reported [3-6].

A healthy 20-year-old man on bupropion for new-onset mood disorder presenting with acute urticaria is described. Delayed onset bupropion-associated urticaria was clinically suspected and his skin lesions resolved with cessation of bupropion and symptomatic management. The clinical features of this rare adverse reaction are also discussed.

## Case presentation

A healthy 20-year-old man without previous history of allergies, angioedema, or other dermatologic conditions presented with a diffuse, severely pruritic erythematous rash all over his body that began two days prior to presentation. He denied any recent environmental exposures or new hygiene products (such as detergents, lotions, or soaps). He had recently been found to have a mood disorder in which he was referred for psychotherapy and started on extended-release bupropion 150 mg daily approximately three weeks ago; he had not associated the relationship between the new medication and the appearance of his rash.

**Figure 1:**
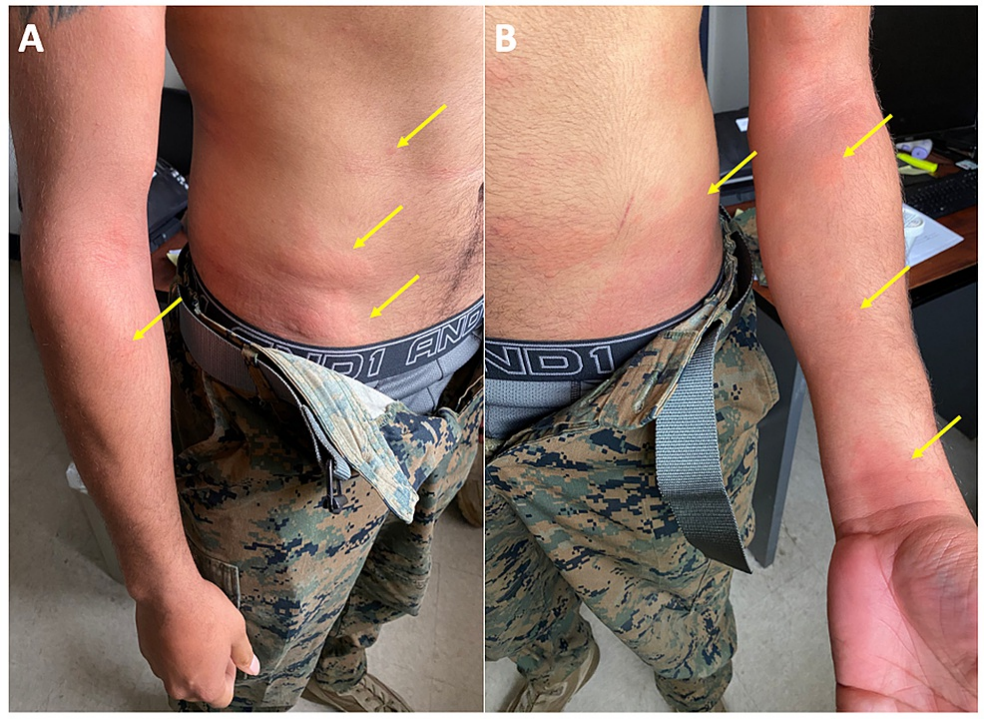
Clinical presentation of delayed onset bupropion-associated urticaria. Right side (A) and left side (B) views of a 20-year-old man with diffuse, scattered erythematous wheals (yellow arrows) on his abdomen, bilateral flanks, and upper extremities that appeared 21 days after bupropion initiation.

Initial assessment showed that his vital signs were within normal limits and he was in no acute distress. Cutaneous examination revealed diffuse, scattered erythematous wheals on his abdomen, bilateral flanks, and upper extremities (Figure 1). Intraoral and cardiopulmonary examination revealed no airway involvement concerning angioedema, and the remainder of the physical examination was unremarkable.

Correlation of the history and clinical features raised suspicion of delayed onset bupropion-associated urticaria; the patient deferred biopsy. He was instructed to discontinue bupropion and was symptomatically managed with oral cetirizine 10 mg daily and diphenhydramine 50 mg in the evening. The skin lesions and associated pruritus significantly improved the following day after treatment initiation and complete resolution of his symptoms and skin lesions was achieved over the following week and antihistamines were discontinued.

Following discontinuation of bupropion and resolution of his skin rash, the patient followed up in the clinic where a care plan for his mood disorder was discussed. Given the severely pruritic rash that followed antidepressant administration, the patient refused a drug re-challenge and alternative medication management; he continued with psychotherapy as the primary treatment modality for his mood disorder. This was a reasonable alternative treatment plan given the recent discovery of his mood disorder without any associated safety concerns; furthermore, he had monthly follow-up appointments to assess for clinical improvement and to discuss the possibility of alternative antidepressant therapy if it was considered necessary.

## Discussion

Bupropion is a dual norepinephrine and dopamine reuptake inhibitor that is approved for the treatment of a major depressive disorder, seasonal affective disorder, and smoking cessation; its off-label use includes the treatment of attention-deficit/hyperactivity disorder, bipolar depression, and sexual dysfunction secondary to antidepressant use. Formulations available in the United States include immediate release, taken twice daily as 75 mg or 100 mg tablets, or extended-release, taken once daily as 150 mg, 300 mg, or 450 mg tablets. The maximum cumulative dose is 450 mg per day in healthy adults and 300 mg per day in pediatric patients; dosing must be adjusted for hepatic or renal impairments [3].

Bupropion is generally well tolerated. Adverse reactions tend to occur at higher doses taken for extended periods of time. Common adverse reactions to the drug may range from being mild (such as agitation, dry mouth, headache, insomnia, and nausea) to severe (such as hypersensitivity reactions, seizures, and suicidality) [3]. Rarely, bupropion may elicit other serious reactions such as anaphylaxis, angioedema, erythema multiforme, generalized exanthematous pustulosis, serum sickness-like reaction, Steven-Johnson’s syndrome, and urticaria [5-12].

Bupropion-associated urticaria is an infrequent adverse reaction, and the estimated incidence is between 1% and 2% with an age and sex predilection favoring men less than 40 years of age. Urticarial rashes have also been observed in individuals who have taken other antidepressants with lesions typically appearing 1-14 days after initiation. In contrast, bupropion-associated urticaria typically appears 15-28 days after the patients begin the medication-a phenomenon very specific to this antidepressant (Table 1) [5].

**Table 1:** Antidepressant-associated urticaria: onset of urticaria after initiation of antidepressants.

Class	Medications	Incidence of urticaria 1-14 days after initiation of drug	Incidence of urticaria 15-28 days after initiation of drug	Reference
Atypical	Mirtazapine and trazodone	3.65%	2.82%	[5]
Monoamine oxidase inhibitors (MOA)	Moclobemide	3.01%	3.56%	[5]
Norepinephrine-dopamine reuptake inhibitors	Bupropion	4.58%	11.98%	[5]
Serotonin-norepinephrine reuptake inhibitors (SNRI)	Duloxetine, milnacipran, and venlafaxine	4.67%	3.57%	[5]
Selective serotonin reuptake inhibitors (SSRI)	Citalopram, escitalopram, fluoxetine, fluvoxamine, paroxetine, and sertraline	3.71%	3.72%	[5]
Tricyclic antidepressants (TCA)	Amitriptyline, clomipramine, dothiepin, doxepin, imipramine, and maprotiline	5.94%	3.89%	[5]

Although unequivocal direct causation of our patient’s urticaria could not be definitively established, bupropion-associated urticaria was strongly suspected based on the history and clinical correlation. In addition, the Naranjo probability scale was used to assess the likelihood for a causal relationship for our patient’s urticaria resulting from an adverse drug reaction to bupropion (Table 2). The scale is based on the total score: less than or equal to zero is doubtful, one to four is possible, five to eight is probable, and greater than or equal to nine is definite. Our patient had a total score of six which correlates with a probable adverse drug reaction to bupropion; however, the score is limited by the fact that the patient’s dose was not changed prior to discontinuing the medication and he was never re-challenged with bupropion [13].

**Table 2:** Naranjo scale questionnaire utilized to estimate the probability of an adverse drug reaction. The probability of an adverse drug reaction is based on the total score and is considered either doubtful (if the score is less than or equal to zero), possible (if the score is one to four), probably (if the score is five to eight), or definitive (if the score is greater than or equal to nine).

Questionnaire	Yes	No	Unknown	Score
1. Are there previous conclusive reports on this reaction?	+1	0	0	+1
2. Did the adverse event appear after the suspected drug was administered?	+2	−1	0	+2
3. Did the adverse event improve when the drug was discontinued or a specific antagonist was administered?	+1	0	0	+1
4. Did the adverse reaction reappear when the drug was readministered?	+2	−1	0	0
5. Are there alternative causes (other than the drug) that could on their own have caused this reaction?	−1	+2	0	+2
6. Did the reaction reappear when a placebo was given?	−1	+1	0	0
7. Was the drug detected in the blood (or other fluids) in concentrations known to be toxic?	+1	0	0	0
8. Was the reaction more severe when the dose was increased or less severe when the dose was decreased?	+1	0	0	0
9. Did the patient have a similar reaction to the same or similar drugs in any previous exposure?	+1	0	0	0
10. Was the adverse event confirmed by any objective evidence?	+1	0	0	0
Total score				6

While the mechanism of delayed onset bupropion-associated urticaria is not yet fully elucidated, it has been hypothesized that the adverse drug reaction is linked to the structure of the drug. Bupropion is structurally similar to amfepramone, a selective norepinephrine releasing agent associated with stress-induced adrenergic urticaria. Alternatively, the patient may have an unknown allergy to one of the ingredients utilized in the tablet formulation of this antidepressant [5,6].

Bupropion has been observed to reduce systemic inflammation. Therefore, this effect of the drug may account for the delayed onset of a bupropion-associated allergic reaction. In contrast to other antidepressants in which medication-induced urticaria presents promptly after starting therapy, urticaria from bupropion typically develops several weeks after initiating the drug [5,6].

A tissue sample biopsy is not required if urticaria can be diagnosed clinically; however, a biopsy may be utilized if the lesions do not resolve with routine care. Management begins with the discontinuation of bupropion. Symptomatic treatment should incorporate histamine H1-blockers such as cetirizine or loratadine taken in the morning and diphenhydramine or hydroxyzine in the evening; additionally, dosage and frequency of antihistamines may be titrated by the managing physician to achieve clinical improvement. For patients with severe hypersensitivity reactions or antihistamine-resistant urticaria, systemic corticosteroids may also be utilized [6].

Once symptoms resolve following an adverse drug reaction, a physician may consider a drug re-challenge if the patient had not experienced a severe adverse reaction, if the clinical benefit outweighs the risk, and if the patient wishes to restart the medication after discussing the associated risks and alternative therapies. A drug re-challenge involves a trial of the medication at a low dose with close monitoring over an extended period; typically, this is completed in the inpatient ward to meet observational requirements. Given that our patient had only been on bupropion for a short period of time in which clinical benefit was limited with no other antidepressants previously trialed and his desire to withhold medication management of his mood disorder, a drug re-challenge was deferred.

## Conclusions

The increased use of antidepressants, secondary to the COVID-19 pandemic, may be associated with an increased incidence of rare adverse drug reactions. Bupropion is a commonly used and well-tolerated antidepressant that is often used to treat various mood disorders. Delayed onset urticaria is an infrequently described adverse reaction that may present weeks after bupropion initiation; clinically, it presents as diffuse, severely pruritic erythematous wheals. Delayed onset bupropion-associated urticaria was clinically suspected in our patient based upon his clinical history and physical examination. Direct causation was not established; however, the Naranjo probability scale was utilized and suggested a probable adverse drug reaction. This rare reaction is usually able to be treated with discontinuation of bupropion and symptomatic management that includes morning and evening oral antihistamines; systemic corticosteroids may be utilized in severe or treatment-resistant cases. A drug re-challenge may be attempted once symptoms resolve if the clinical benefit outweighs the risk and the patient is agreeable after discussing associated risks and available alternative therapies.
